# Time Series and Pattern of Dog Bites and Rabies in the Ashanti Region, Ghana, 2009–2019: A Call for Inter-Sectoral Collaboration in the Fight Against Rabies

**DOI:** 10.3390/tropicalmed11090260

**Published:** 2026-09-20

**Authors:** William Tasiame, Sherry Ama Mawuko Johnson, Obed Owusu Yeboah, Benjamin Emikpe, Raphael Folitse, Vitus Burimuah, Theophilus Odoom, Christian Drosten, Victor Max Corman, Philip El-Duah

**Affiliations:** 1 School of Veterinary Medicine, Kwame Nkrumah University of Science and Technology, Kumasi P.O. Box 256, Ghana; drwilly2002@gmail.com (W.T.); banabis2001@yahoo.com (B.E.); raphfolitse@yahoo.com (R.F.); burimuah@gmail.com (V.B.); 2 Institute of Virology, Charité–Universitätsmedizin Berlin, Corporate Member of Freie Universität Berlin and Humboldt-Universität zu Berlin, Charitéplatz 1, 10117 Berlin, Germany; 3 School of Veterinary Medicine, College of Basic and Applied Sciences, University of Ghana, Legon, Accra P.O. Box LG139, Ghana; sherryjohnson03@gmail.com; 4 Veterinary Services Department, Ministry of Food and Agriculture, Accra P.O. Box M161, Ghana; kingobed91@gmail.com; 5 Accra Veterinary Laboratory, Veterinary Services Directorate, Accra P.O. Box M161, Ghana; theodoom@gmail.com

**Keywords:** rabies, dog, disease surveillance, public health, Ghana, One Health

## Abstract

Dog-mediated human rabies is a vaccine-preventable neglected zoonotic disease, causing thousands of deaths every year across Africa and Asia. Information to generate the appropriate attention and action to avert human rabies is lacking due to disaggregation of available data. This study sought to understand the burden of dog-mediated rabies transmission and response patterns in Ghana’s Ashanti Region, and analyzed dog-bite surveillance, rabies laboratory investigations, and hospital visitation data from 2009 to 2019. A total of 442 bite incidents were recorded, with most occurring in the Kumasi metropolis, particularly in the Oforikrom and Manhyia sub-metros. Many victims sought healthcare outside their bite-incident districts, often at the Manhyia Government Hospital, likely due to vaccine availability. Bite incidents were generally reported promptly within two days, although final investigations took about fourteen days. The sale of implicated dogs for human consumption during observation periods posed an additional rabies risk. There was a 67.53% positivity for animal rabies (*n* = 156/231) in laboratory investigations, and dogs accounted for most confirmed cases (141/210), though livestock and non-human primates were also affected. More than 70% of dog-bite victims were linked to rabies-positive animals, showing a significant association between bites and confirmed rabies cases. Reported dog-bite frequency varied considerably over time, with the highest monthly frequencies occurring between 2010 and 2013, and with a sustained positive seasonal effect from August to November within a year. The findings highlight that dog-mediated rabies remains a major public health threat in the region and emphasize the need for improved One Health investigations, surveillance, and public education to reduce rabies transmission and exposure risks.

## 1. Introduction

Rabies remains a neglected viral zoonotic disease affecting poor rural communities in most of the developing world in Africa and Asia. Although the disease is preventable through vaccination of dogs and the use of post-exposure prophylaxis (PEP) for exposed persons, approximately 60,000 human deaths are estimated to occur each year worldwide [1,2]. Rabies mortality is unparalleled among zoonotic diseases; moreover, once clinical disease develops, it is almost invariably fatal, resulting in an exceptionally high case-fatality rate [3,4]. Bites from infected rabid dogs account for more than 99% of human rabies cases [5,6]. Children below 15 years of age mostly bear the brunt of dog bites, which could result in rabies [7], severe trauma and disfigurement, psychological issues and other dog-related zoonotic infections [8,9]. On average, the incubation period for rabies in humans ranges from 2 to 3 months; however, factors such as the extent and proximity of the wound to the nervous system, the inoculum size, and the viral strain may influence the duration and clinical course of the disease [10,11].

Available sensitive surveillance protocols play a pivotal role in decision-making for the control and management of diseases [12]. In Africa, where the burden of rabies remains substantially higher than in Europe and North America [13], underreporting, inconsistent data, and low political will have partly contributed to the persistence of the disease [14]. Furthermore, the glaring inability of stakeholders from the human and animal sectors to harmonize data and cumbersome, disengaged reporting systems from lower to national levels promote disregard for the needed attention rabies deserves [15]. Community animal rabies data linked to health institutions is a sure way to demonstrate possible integration of rabies information as a priority disease worth national attention [16]. Furthermore, dog-bite data are sometimes the only useful epidemiological evidence clinicians rely on to commence the limited and expensive PEP for exposed persons [17].

Ghana records a considerable number of animal and human rabies cases, with associated fatalities. For instance, during a single cross-border canine rabies outbreak in Burkina Faso and Bongo in the Upper East Region of Ghana, nine persons exposed to rabies-infected dogs died [18]. The occurrence of rabies in Ghana also extends beyond humans and dogs, with cases reported in farm animals such as pigs, goats, and rabbits [19,20]. Rabies diagnosis in Ghana remains constrained, with animal cases diagnosed in only a few laboratories using the Sellers stain and direct fluorescent antibody test, while human cases are diagnosed mainly based on clinical symptoms. These diagnostic limitations are compounded by inadequate human–veterinary collaboration, inadequate diagnostic facilities, and prevailing cultural beliefs [21]. The persistent burden of rabies in Ghana may be attributed to several interrelated factors, including a high free-roaming dog population, erratic and unfocused dog vaccination, knowledge gaps, poverty, poor surveillance, and, to some extent, cultural practices [22]. Globally, the inadequacy and fragmented nature of rabies surveillance data that contribute to doubts about the true impact of rabies drive the diminished capacity to advocate for public health support [23]. Providing well-synthesized, region-specific information in this regard will provide a basis for advocacy for tailored support geared towards the elimination of rabies. To this end, this study sought to elucidate rabies surveillance data to incite discussion on gap-filling solutions.

## 2. Materials and Methods

### 2.1. Study Area

The Ashanti Region is situated in the Middle Belt of Ghana, spanning an area of 24,389 square kilometers, representing 10.2% of the total surface area of the country [24]. The region had an estimated 2025 human population of 5.8 million, and has 121 hospitals, 103 health centers, 282 clinics, and 133 Community-based Health Planning and Services (CHPS) compounds [25]. According to the Ghana Veterinary Services Directorate (VSD) animal census estimates of 2018, the Ashanti Region had 190,678 dogs, which represented 20.9% of recorded dogs in the country and was the highest among the 10 administrative regions in Ghana at the time (VSD data).

### 2.2. Data Collection and Analysis

A region-based, retrospective, observational study using secondary data was performed to determine the occurrence of dog bites and laboratory investigations, hospital visitation patterns, and the time course of incident reporting to case resolution from 2009 to 2019. For this, unstructured data were retrieved from the Ashanti Regional Veterinary Clinic and Laboratory located in Kumasi on 17th January, 2020. Data were cleaned in Microsoft Excel and analyzed using R statistical package version 3.6.3 (R Core Team, Vienna, Austria). Maps showing the distribution of bites and hospitals visited were generated using Quantum GIS version 3.6.2 and data freely available from diva-gis.org and www.gadm.org. Descriptive statistics were performed using bar charts and boxplots, and inferential statistics were performed using chi-square analysis and a Wilcoxon rank-sum test. The rabies incidence rate was estimated using the Ashanti Region dog population, and the 95% confidence intervals for the incidence rates were calculated by the Poisson exact method, using OpenEpi software version 3.01. A Poisson regression analysis was also performed to evaluate the predictive capacity of dog rabies positivity for the number of bite victims using JASP statistical software version 0.98.1.

### 2.3. Time-Series Analysis of Dog Bites

To analyze the long-term trends and short-term seasonal dynamics of dog-bite frequencies over the 11-year study period, a time-series forecasting framework was utilized, specifically a Bayesian Generalized Additive Model (Facebook Prophet), in JASP software version 0.98.1.

The dataset, consisting exclusively of days featuring recorded dog-bite incidents, was aggregated to a monthly frequency, yielding a continuous timeline of 132 observations. This aggregation strategy was intentionally selected over daily modeling to smooth binary low-count noise (predominantly 0 and 1 incident counts), thereby ensuring continuous distribution curves. Chronological continuity was maintained by imputing a value of zero for months experiencing no documented incidents.

To evaluate the impact of local macro-climatic cycles on canine and human behavior, three mutually exclusive weather regimes were defined based on the regional calendar: rainy season, minor rainy season, and dry season (Harmattan). Due to distinct localized weather anomalies characterized by a temporary suspension of precipitation within the primary monsoon cycle, August was isolated from the general rainy season and modeled independently as a standalone “August Interlude” covariate. The August Interlude was systematically omitted from active model inputs to serve as the baseline reference control group.

The Bayesian Prophet model was deployed to decompose the monthly time series into structural components consisting of a long-term growth trend, annual seasonality, and external regressors. To isolate major macroeconomic structural shifts and prevent overfitting to random monthly fluctuations, the maximum number of allowable trend changepoints was restricted to a threshold of 11 (representing approximately one potential shift per study year) across an active data window of 80% (0.8). The trend flexibility was governed via a Laplace prior scale distribution.

For the Bayesian Prophet model, the statistical reliability of the parameters was evaluated using 95% credible intervals, where an effect was deemed robust if its interval did not transcend zero. The visual stability of the long-term trend and the annual seasonal waves was confirmed via component decomposition plots.

## 3. Results

### 3.1. Data Collected and Case Distribution

**Figure 1:**
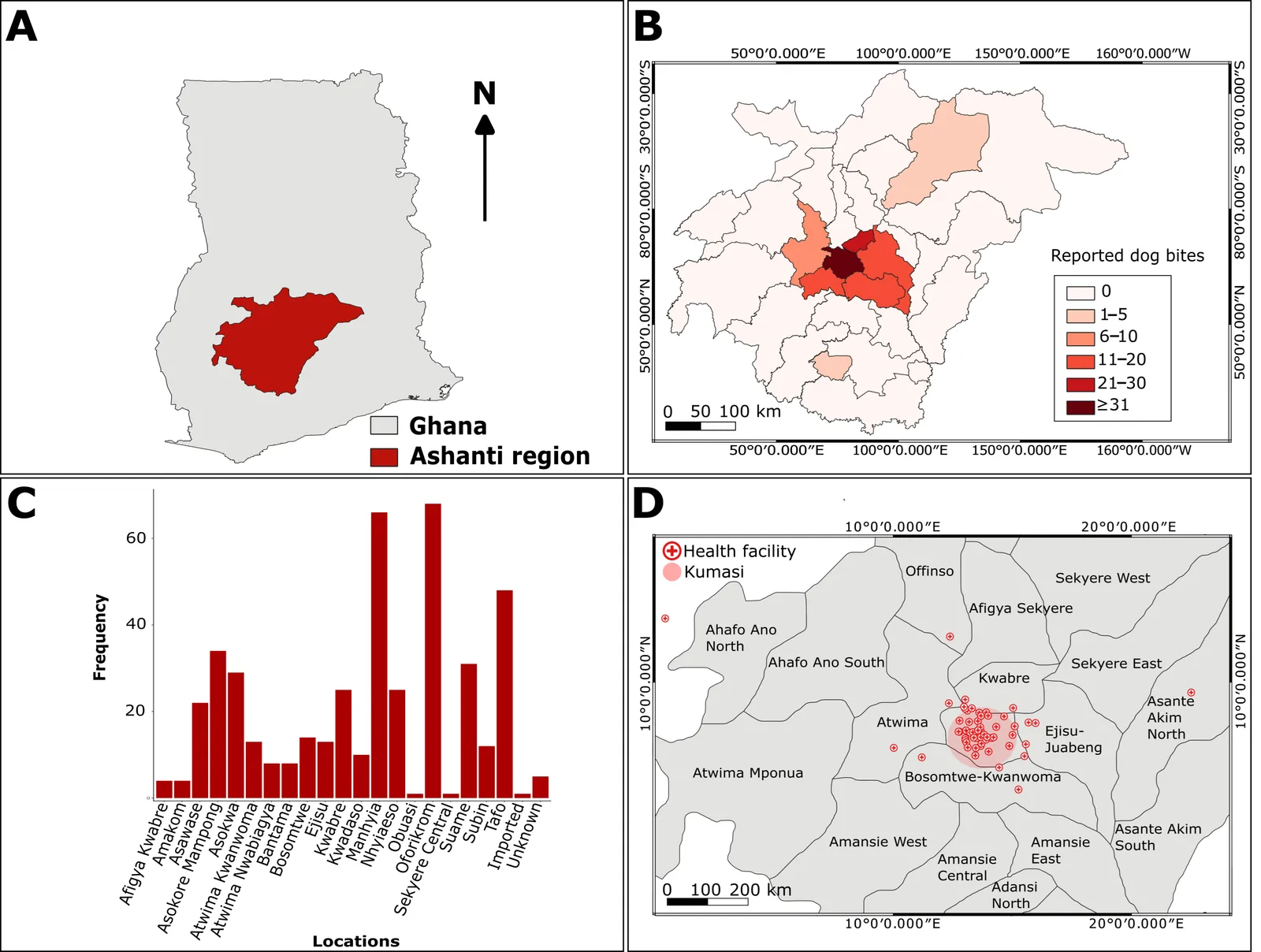
Distribution of reported dog-bite cases and hospitals visited in the Ashanti Region of Ghana. Panel (**A**) is a map of Ghana showing the Ashanti Region. Panel (**B**) is a map of the Ashanti Region showing the distribution of dog bites in the various districts of the Ashanti Region. Panel (**C**) depicts the distribution of dog-bite cases in the Kumasi metropolis, and panel (**D**) shows the distribution of hospitals visited within the Ashanti Region. Maps were generated with Quantum GIS version 3.6.2 and data freely available from diva-gis.org and www.gadm.org.

The data obtained represented all bite incidents in the Ashanti Region of Ghana (Figure 1A) reported to the Amakom Government Veterinary Clinic located at the Regional Veterinary Directorate, Kumasi. A total of 442 complete records of bite incidents reported in the region within the 11-year period were analyzed, with a higher number of reports around the regional capital and fewer reported cases outside the capital, Kumasi. All the implicated dogs were technically owned per records; however, there were an additional 42 bite incidents without information on ownership and location of the incident, which were excluded from the analysis. The lack of information on ownership could indicate ownerless dogs; however, this cannot be said for certain. In Ghana, owned dogs can still be free-roaming [26]; therefore, the confinement status of these dogs could not be inferred from this data as well. No additional information on the bite incidents was available from the data. There was one reported incident each from the Obuasi and Sekyere Central municipalities, representing 0.2% each of the total reported incidents, and an additional eight (1.8%) incidents reported from the Atwima Nwabiagya municipality. Comparable numbers of bites were reported from the Atwima Kwanwoma and Ejisu Juaben municipalities, both with 13 (2.9%), together with the Bosomtwe district (n = 14, 3.2%). The Kwabre district recorded 25 (5.7%) bite incidents, and the Kumasi metropolis recorded 367 (83%), which was the highest number of reported incidents (Figure 1B).

Within the Kumasi metropolis, the highest numbers of dog-bite incidents were reported from the Oforikrom (n = 68, 15.4%) and Manhyia (n = 66, 14.9%) sub-metros (Figure 1C). Like bite incidents, hospitals visited due to dog bites were concentrated around the Kumasi metropolis (Figure 1D). A total of 422 hospital visits were made to 58 different healthcare facilities as a result of dog bites within the time frame under study. A smaller proportion of dog-bite victims visited hospitals within the same district where the bite occurred (n = 156, 37%), while 266 (63%) visited a healthcare facility outside the district of the bite incident. The majority of the hospital visitations outside the district of the bite were to the Manhyia Government Hospital (n = 105, 39.5%), which is the International Vaccination Centre of the Ashanti Region of Ghana [27]. The median frequency of visitation per healthcare institution was, however, not significantly different between the groups that visited healthcare facilities within and outside the district of the dog bite based on a Wilcoxon rank-sum test (*p* = 0.05319).

### 3.2. Time Course of Bite-Incident Reporting and Clinical Outcomes

**Figure 2:**
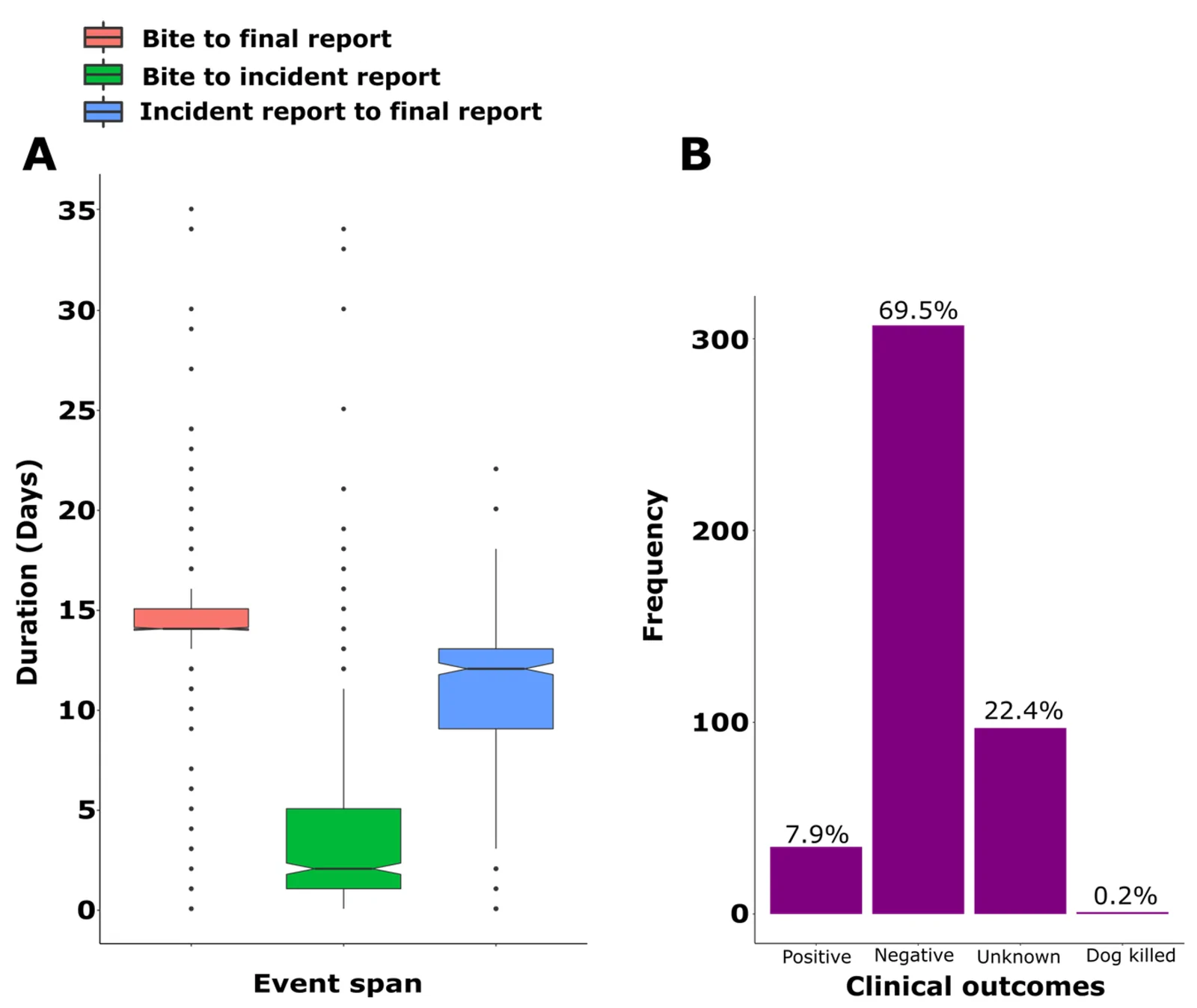
Time course of bite-incident reporting and outcomes of clinical investigation. Panel (**A**) shows the distribution of duration for case reporting by dog owners and report issuance by the Veterinary Services Department in Kumasi. Panel (**B**) depicts the distribution of clinical outcomes of cases determined by the veterinary services after observation periods.

The shortest duration between a dog bite and an initial report to the veterinary services was on the same day, with a median duration of 2 days and a maximum of 34 days. The minimum duration between a bite incident and the issuance of a final report by the veterinary services was on the same day, with a median duration of 14 days and a maximum of 35 days. The minimum duration between an incident report and the issuance of a final report by the veterinary services was also on the same day, with a median duration of 12 days and a maximum of 22 days (Figure 2A). Based on an established 14-day observation criterion used by the Ghana Veterinary Services after a bite incident, 307 (69.8%) cases were clinically determined to be rabies-negative and 35 (8%) rabies-positive. The 35 cases were identified over 2,097,458 dog-years at risk (dog population multiplied by years studied), resulting in an overall incidence rate of 1.67 cases per 100,000 dogs per year (95% CI: 1.16, 2.32). The determination of rabies positivity is based on the development of classical rabies symptoms such as salivation, loss of appetite, or fear of water during the observation period. The clinical outcome of 99 (22.4%) cases was unknown as a result of lack of feedback from the incident reporter on the status of the dog within the 14-day observation period after the initial report was made. One person said the dog was killed within the 14-day period, most likely as a result of aggressive behavior shown by the dog (Figure 2B).

To obtain an overview of cases with unknown clinical outcomes, follow-up calls were made to all incident reporters from February, 2018, to December, 2019. A total of 27 calls were made, of which no contact was established in 10 (37%) instances. A further 10 people reported that the implicated dogs were alive, which points to the absence of rabies in these dogs. Six people (22.2%) reported that the dogs had died, and one reported that the dog went missing (Table 1). Among the dogs that had died, four persons reported that this occurred within the 14-day observation period, while one person said this occurred after the 14-day period. Reported causes of death were acute enteritis (1, 16.67%) and being sold for slaughter and consumption (4, 66.67%), as seen in Table 1.

### 3.3. Time-Series Assessment of Dog-Bite Incidents

**Figure 3:**
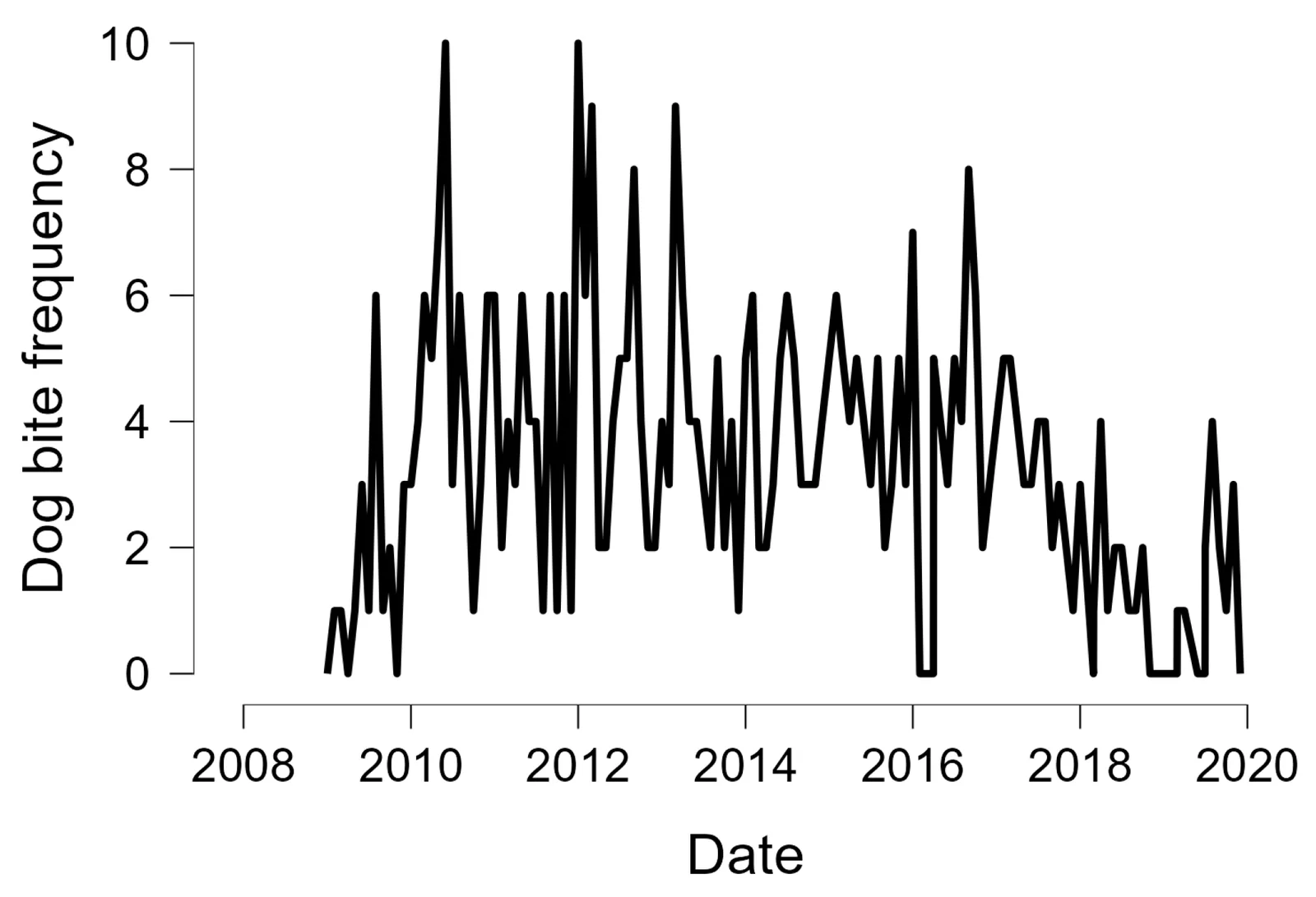
Historical monthly time-series plot of reported dog-bite frequency, 2009–2019. The figure depicts the observed distribution of monthly dog-bite frequency, showing substantial temporal variability over the observation period.

Reported dog-bite frequency varied considerably over time, with the highest monthly frequencies occurring between 2010 and 2013 (up to 10 bites/month). Frequencies remained relatively elevated but highly variable through approximately 2017, followed by a general decline from 2017 onwards and several months with zero or very low reported bites during 2018–2019 (Figure 3).

**Figure 4:**
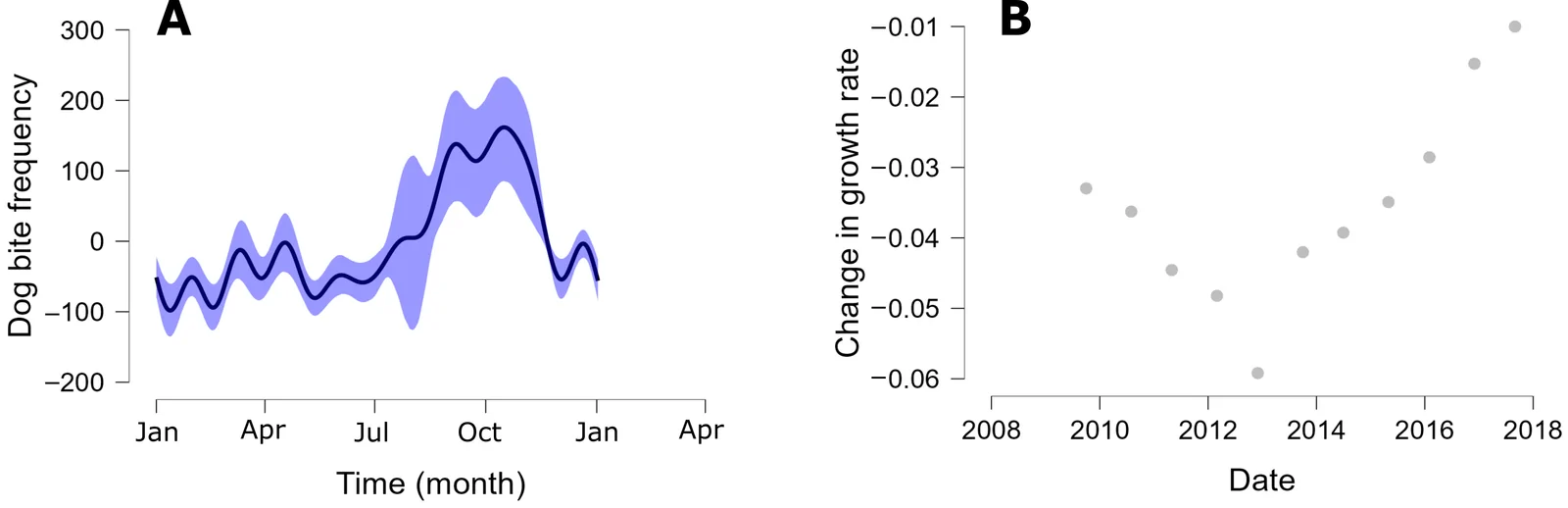
Seasonality and changepoint analysis of reported dog-bite frequency. Panel (**A**) depicts a seasonality plot; the *Y*-axis shows the additive effect (the baseline adjustment) for that specific time of year, not raw dog-bite frequencies, and the *X*-axis shows the time in calendar months. The black trend line indicates the mean estimate and represents the most likely seasonal path of dog bites across the 12 months. The purple band around the solid line represents the uncertainty interval (95% credible interval) for the seasonal trend. The model is less certain about the exact seasonal effect because some years had huge spikes while others had deep drops during those calendar months (August to November). Panel (**B**) depicts the change in growth rate (*y*-axis) plotted against the date (*x*-axis, 2008–2018). There are 11 chronological points tracing a clear V-shaped (trough) trajectory. The changepoint analysis shows the rate of decline in dog bites, not a switch from growth to shrinkage.

The model initialized with a baseline growth rate of 0.2 (95% credible interval: 0.07 to 0.42), which represents a statistically significant baseline trend of accelerating dog-bite incidents at the start of the study window (2009–2010). The estimated yearly seasonality plot displays a distinct cyclical wave partitioned by the calendar halves. The first seven months of the year (January to July) exhibit a consistent negative seasonal effect, suppressing bite frequencies below the baseline. Conversely, the second half of the year (August to November) demonstrates a sustained positive seasonal effect, accelerating bite frequencies and peaking in the latter third of the year, but dropping again in December below baseline and with some credible intervals transcending zero (Figure 4A). Across the observed period, all estimated changepoint effects were negative, implying that the dog-bite series was in overall decline throughout. The most pronounced negative changepoint was estimated around 2013, indicating the steepest decline at that time. However, because the 95% credible intervals for all changepoints included 0, we cannot conclude with high confidence that there was a statistically reliable change in the trend; the 2013 observation should therefore be viewed as a plausible but uncertain inflection point (Figure 4B).

### 3.4. Laboratory Testing, Species Implication, and Human Involvement

**Figure 5:**
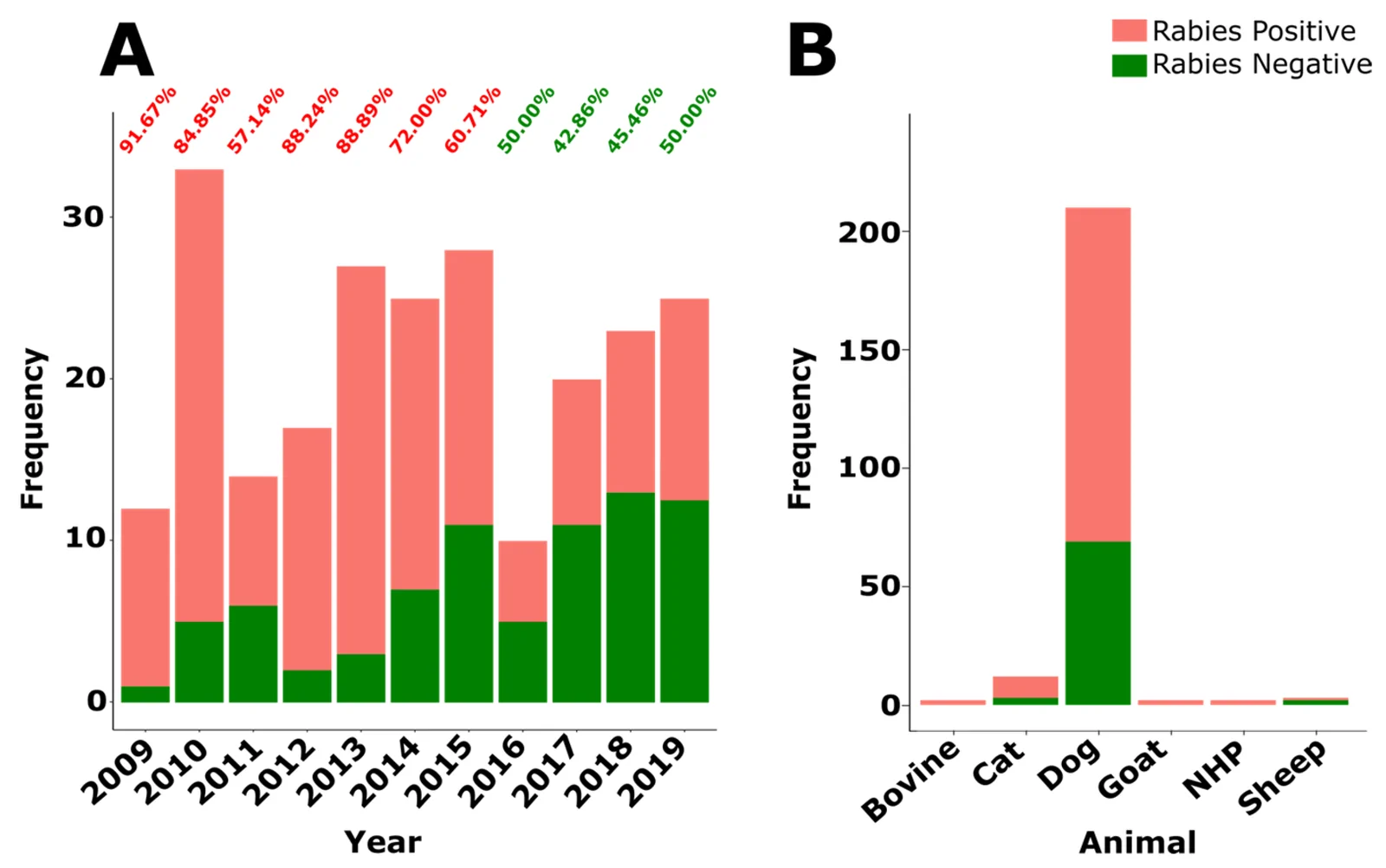
Distribution of laboratory outcomes of cases per year and cases per species. Panel (**A**) shows the distribution of cases, laboratory testing outcomes, and positivity rates for rabies cases from 2009 to 2019. Percentages in red represent positivity rates above 50%, and those in green represent positivity rates of 50% and below. Panel (**B**) depicts the distribution of laboratory testing outcomes of rabies cases reported in various animal species. The animal designation NHP stands for non-human primate.

A total of 231 laboratory cases were recorded within the period of study, of which all tests performed prior to January 2019 were done by the Sellers staining approach, and the fluorescent antibody test was used after this time. The highest number of samples sent to the laboratory for rabies testing was recorded in 2010 at 33, and the lowest was recorded in 2016 at 10. The highest proportion of positivity was recorded in 2009, with 11 out of 12 cases testing positive (91.67%). In 2016, there was an equal number of positive and negative cases, but prior to 2016, there were always more than 50% positive cases among the samples tested (57.14–91.67%) compared to the period from 2016 (42.86–50%) (Figure 5A). There was a statistically significant association between the year in which laboratory outcomes were reported and rabies positivity based on a chi-square test of independence (*X*^2^ (10, N = 231) = 33.36, *p* < 0.001). Over 70% (n = 128) of human victims of dog bites were bitten by rabies-positive animals. A chi-square test of independence showed a significant association between animal rabies positivity and biting humans (*X*^2^ (1, N = 231) = 4.2, *p* = 0.0307), and a Poisson regression analysis showed that dog rabies positivity was a significant predictor of the number of bite victims (B = 0.30, SE = 0.14, z = 2.07, *p* = 0.04). The corresponding Incident Rate Ratio (IRR = 1.35) showed rabies-positive dogs to have a 35% higher rate of bite victims compared to rabies-negative dogs.

In total, 156 (67.53%) animals were positive for the rabies virus. Animals implicated in rabies cases were cattle (n = 2, 0.87%), cats (n = 12, 5.19%), dogs (n = 210, 90.91%), goats (n = 2, 0.87%), sheep (n = 3, 1.30%), and two non-human primates (0.87%). Proportions of 67.14% (n = 141) of dogs, 75% (n = 9) of cats, 33.33% (n = 1) of sheep, 50% (n = 1) of goats and non-human primates, and 100% of cattle tested positive by laboratory investigation (Figure 5B).

## 4. Discussion

Accurate rabies datasets constitute one of the fundamental stepping stones for successful rabies control programs. Unlike in Europe and America, endemic rabies countries in Africa lag behind in this regard; thus, passive surveillance reports could help address part of this information gap [28]. Our study found that the majority of bite incidents were reported to veterinary or health institutions situated in the capital of the Ashanti Region, Kumasi. Capital cities are usually endowed with amenities such as hospitals, good roads, and radio and television stations, facilitating easy access to increased information and use of these facilities. A study from Ethiopia posits similar findings of higher reports from Addis Ababa, the capital city [29]. Even though rabies affects more rural communities located in the outskirts, control and prevention programs are most often organized in urban areas, possibly enhancing reports at the detriment of rural areas where the situation may be more pronounced [30].

Our findings indicated low submission and positivity of suspected rabies cases in 2016; however, this started increasing in subsequent years. The Ghana Veterinary Service has been bedeviled with a shortage of staff over the years as retirements increased. This was exacerbated by the ban on the employment of technical veterinary staff by the Ghana government over the years. It was not until 2010 that two veterinary medical schools began taking students. The first batch graduated in 2016, with a resultant influx of newly trained veterinary officers into the service. These reasons could affect the interpretation of laboratory results, especially in the case of the Sellers staining technique, which requires highly trained personnel with experience to accurately view the Negri bodies. The need for newer, more accurate rabies diagnostic equipment that does not depend on the person performing the test represents a priority for the future.

High visitations were recorded in Manhyia and Oforikrom, which have the Manhyia and KNUST Government Hospitals, respectively, in the capital. Cost is known to play a major role in hospital attendance; consequently, more people sought care at government hospitals in this study [17,31]. In our study setting, Manhyia Government Hospital serves as the International Vaccination Centre in the Ashanti Region, suggesting that the availability of rabies vaccination may also have contributed to increased attendance for bite exposures [27]. In contrast to our findings, another study reported higher hospital visitation for bite exposures among rural residents [32].

Dog-bite exposures constitute a major public health concern and threat to rabies transmission, especially in rabies-endemic regions [33,34], hence the urgent need to notify veterinary authorities to initiate investigation. Our finding of an average of 2 days spent from a bite to initial notification was within an acceptable time by which investigations could be initiated, and the risk of developing rabies was low. This gives ample time for veterinarians to follow up on culprit biting dogs and offer appropriate advice to victims. Similarly, the majority of the outcomes were within the 14-day period used in Ghana by reporting veterinarians to notify physicians on the status of dogs for possible PEP. Even though the median incubation period to develop human rabies post-exposure from infected animals is usually 1 to 2 months, cases with clinical manifestations within a week of the bite have been reported [35,36,37]. Such short incubation periods may substantially reduce the window for recognizing the exposure and initiating timely post-exposure prophylaxis, underscoring the importance of immediate wound management and prompt access to PEP. Of note, the 22.4% incident case without a conclusive outcome or a final follow-up after the 14-day observation period is a potential risk for rabies transmission in the event of a positive outcome unknown to the veterinary clinic. Further enquiries from these inconclusive outcomes indicated that some owners sold dogs to butchers for human consumption to get rid of biting dogs and make some money. This information is corroborated in a study from Ghana which demonstrated rabies virus RNA in dogs slaughtered for human consumption [38] as well as in other studies [39,40,41], highlighting the potential risk for rabies transmission from butchering and consumption of dogs. The potential risk should not be interpreted as evidence that consumption of dog meat itself directly increases rabies transmission but rather that it increases exposure across different stages of the associated value chain, particularly through the handling and processing of dogs.

The positive baseline growth rate seen in the time-series analysis indicates that, at the start of the time period under study, bite frequencies were accelerating, potentially driven by increasing exposure, reporting, or other underlying risk in the late 2000s. The negative changepoint around 2013, which was not statistically significant, signals a gradual macroeconomic wave rather than a sharp, sudden crisis. This could, for instance, be linked to transformations that occurred in how Ghana tracked public health data affecting dog bites, such as the rollout of the District Health Information Management System (DHIMS2) from 2012 and the start of the entry of dog bites and rabies cases into the system beginning in 2013 by the Ghana Health Service (GHS) [42]. The observed trend in growth rate is likely an artifact of transitioning from a fragmented, paper-based system to an electronic system rather than an actual change in dog-bite frequencies. The rate of reporting of new cases slowed down even though total counts kept rising, most likely because the digital system eliminated double counting, filtered out duplicate entries, and stopped the bulk entry of historical backlogs.

The inclusion of dog bites into the DHIMS is a very important change that occurred in reporting practice, which improved inter-sectoral harmonization and integrated surveillance of dog-bite incidents. This improvement, however, still does not translate into the full integration of reporting of veterinary rabies or vaccination status of specific dogs involved in bite incidents [18,43], and can be improved for overall better management of rabies cases. In this regard, further inter-sectoral collaboration in data harmonization is essential.

The marked seasonality, with suppressed bites in the first seven months and elevated risk in the latter third of the year, mirrors patterns reported elsewhere in West Africa, where climatic and social factors like agricultural cycles may concentrate exposures in specific months. In several regions, peak dog-bite incidents align with rainy periods (such as March to May or October to November in parts of East Africa). These times often coincide with female dogs going into heat, triggering intense competition, aggressive behavior, and territorial defense among male dog packs [44,45].

The fact that some credible intervals transcended zero and therefore suggest no statistically significant associations between dry, rainy, or minor rainy season conditions and bite counts in the analysis suggests that, in this setting, seasonal categorizations may not be enough to capture the environmental drivers of dog bites, and more specific variables like temperature and precipitation or non-climatic determinants like dog population dynamics, urbanization, or reporting practices may play more decisive roles [46,47].

Together, these findings underscore the importance of sustaining and targeting prevention efforts toward high-risk periods later in the year, while also highlighting the need for more granular data on both environmental exposures and dog population management to better understand and predict fluctuations in bite incidence in the Ashanti Region.

Rabies diagnostic laboratory support cannot be overemphasized [48]; however, resource-limited endemic countries continue to rely on the Sellers stain technique, a method known for low sensitivity compared to the gold-standard method, the direct fluorescent antibody test [49,50,51] and direct rapid immunohistochemistry test [52]. Our study found the Sellers stain to be the method of choice for a 10-year period, which could affect the proportion of positive samples over the period due to expertise in test performance. Nevertheless, the obvious vector of rabies transmission in Africa, the domestic dog, represented the bulk of cases as well as positives, followed by cats, as seen in other studies in Africa [53,54]. In contrast, wildlife rabies is more pronounced in countries in the American continent [55], with rabies in cats and livestock exceeding that reported in dogs [56]. Although livestock rabies is not prominently reported in Africa, the rippling devastating effect on poor households whose livelihood depends on livestock represents a major concern. It is worth noting that these infected livestock are sometimes processed and consumed by community members, which is a potential source of rabies transmission [57,58].

Aggression resulting in bites, whether provoked or unprovoked, constitutes a public health concern and a probable cause of rabies [59]. Considering the fact that most of the offending dogs in our study were laboratory-confirmed as having rabies, this suggests that human involvement in bite exposures constitutes highly probable cases of rabies in the absence of rabies post-exposure prophylaxis. This aligns with other studies that attributed bite exposures as potential sources of rabies infection [60,61]. In addition, approximately two-thirds of all rabies cases exhibit furious-rabies manifestations, depicting aggression and bites [62,63]. This finding may partly reflect selection bias, as dogs that exhibit aggressive behavior and bite humans are more likely to be presented for rabies investigation, whereas dogs with non-aggressive manifestations are less likely to. Contrary to our study, a rabies-positive outcome was not associated with aggressiveness and bite exposures in another study in the US, which found dogs with the paralytic type of rabies probably originating from wildlife rabies variants [64].

The present study was designed as a retrospective analysis of historical trends in dog bites and rabies rather than as an assessment of the current epidemiological situation, hence the reason the data collected ended in 2019. Consequently, the 7-year period following the study period was outside the scope of the present analysis. Nevertheless, the findings provide important historical evidence on the patterns of dog bites and rabies and may serve as a useful baseline for comparison with more recent surveillance data.

## 5. Conclusions

Our study established that dog-mediated rabies remains a major public health threat, and bite exposures are distinctly associated with rabies in the Ashanti Region of Ghana. Even though a few livestock were infected, affected farmers, who are mostly poor, lost their much-needed income. Although bite-exposed victims reported fairly on time for commencement of investigations, inconclusive outcomes due to lack of follow-up and sale of biting dogs for human consumption constitute a potential risk for rabies in the absence of PEP. The observation of a seasonal pattern of dog bites and disjointed reporting calls for tailored interventions and more inter-sectoral collaboration. We recommend that the veterinary section develop innovative ways to complete all dog-bite reports, beginning with telephone calls, while education to the public needs to be intensified. The interpretation of these findings is limited by incomplete records and the lack of contextual data, which may have constrained a more comprehensive understanding of the observed trends.

## Figures and Tables

**Table 1:** Outcomes of follow-up of unresolved dog-bite cases from 2018 to 2019.

Clinical Question	Response	Frequency (%)
	Alive	10 (37.04)
	Dead	6 (22.22)
Status of the animal	No contact	10 (37.04)
	Went missing	1 (3.70)
	Total	27 (100)
**Details of death (n = 6)**		
	Within observation time	4 (66.67)
Period death occurred	Outside observation time	1 (16.67)
	Unknown	1 (16.67)
	Acute enteritis	1 (16.67)
Cause of death	Sold for slaughter and consumption	4 (66.67)
	Unknown	1 (16.67)

## Data Availability

The original contributions presented in this study are included in the article/Supplementary Materials. Further inquiries can be directed to the corresponding author.

## Supplementary Materials

The following are available online at https://www.mdpi.com/article/10.3390/tropicalmed11090260/s1.

## Abbreviations

The following abbreviations are used in this manuscript:

CHPS	Community-based Health Planning and Services
DHIMS	District Health Information Management System
GHS	Ghana Health Service
GIS	Geographic Information System
KNUST	Kwame Nkrumah University of Science and Technology
PEP	Post-Exposure Prophylaxis
RNA	Ribonucleic Acid
VSD	Veterinary Services Department
